# ASO Author Reflections: Challenges in the Management of Gastroesophageal Junctional Adenocarcinoma

**DOI:** 10.1245/s10434-021-10397-0

**Published:** 2021-08-14

**Authors:** Sivesh K. Kamarajah, Alexander W. Phillips, Sheraz R. Markar, Ewen A. Griffiths

**Affiliations:** 1 Department of Upper Gastrointestinal Surgery, Queen Elizabeth Hospital Birmingham, University Hospitals Birmingham NHS Trust, Birmingham, UK; 2 Institute of Cancer and Genomic Sciences, College of Medical and Dental Sciences, University of Birmingham, Birmingham, UK; 3 Northern Oesophagogastric Unit, Royal Victoria Infirmary, Newcastle University Trust Hospitals, Newcastle-Upon-Tyne, UK; 4 School of Medical Education, Newcastle University, Newcastle upon Tyne, Tyne and Wear UK; 5 Department of Surgery and Cancer, Imperial College London, London, UK; 6 Department of Molecular Medicine and Surgery, Karolinska Institutet, Stockholm, Sweden

## Past

Over the past decade, there has been a marked increase in the prevalence of gastroesophageal junctional (GEJ) cancers, largely attributed to the rising rates of obesity and chronic gastroesophageal reflux disease. A current dilemma in the management of these patients, especially Siewert II GEJ cancers, remains the choice of surgical approach between either a transthoracic esophagectomy or an extended total gastrectomy. This choice remains a difficult one to ensure clear surgical margins are achieved and a radical lymphadenectomy is appropriately performed. While the benefits of performing an esophagectomy includes a radical resection with surrounding mediastinal lymph nodes, it is associated with an increased risk of respiratory complications and may compromise the gastric margin. On the other hand, extended total gastrectomy, performed via a transhiatal or left thoracoabdominal approach, may not compromise the gastric margin, but at the risk of compromising the proximal (esophageal) margin. Although some surgeons may use intraoperative frozen section histology to assess for the threatened margin, this is not always accurate. To date, the evidence to guide clinical practice is limited to retrospective case series^1^,^2^ and is therefore weak to help with decision making.

## Present

The present study^3^ included patients with non-metastatic Siewert II GEJ adenocarcinoma receiving either esophagectomy (*n* = 999) or total gastrectomy (*n* = 8595), from the National Cancer Database (NCDB) from 2010 to 2016. This national population-based cohort study from the US demonstrated that patients receiving esophagectomy had significantly longer survival than total gastrectomy for Siewert II GEJ adenocarcinoma. In an unmatched cohort, patients receiving gastrectomy had significantly lower overall survival than esophagectomy (median: 47 vs. 68 months, *p *< 0.001; 5-year survival: 45% vs. 53%). Following matching, gastrectomy was associated with significantly reduced survival compared with esophagectomy (median: 51 vs. 68 months, *p *< 0.001; 5-year survival: 47% vs. 53%), which remained on adjusted analyses (hazard ratio 1.22, 95% confidence interval 1.09–1.35, *p *< 0.001). The rates of margin-negative resections were significantly higher with esophagectomy than gastrectomy; however, there were no significant differences between postoperative morbidity and mortality and lymph node harvest results between these two approaches. In this large-scale population study with propensity matching to adjust for confounders, esophagectomy was prognostically superior to gastrectomy for the treatment of Siewert II GEJ adenocarcinoma, despite comparable lymph node harvest, length of stay, and 90-day mortality.

## Future

Moving forward, there are several questions to be addressed. First, the extent of lymphadenectomy required to achieve the best oncological outcome remains a dilemma. In patients where staging suggests definite mediastinal nodes, understandably an esophagectomy with two-field lymphadenectomy is likely to confer the greatest survival advantage. However, in patients where lymph node metastases may not be apparent during clinical staging, along with further disease behavior and the spread of micrometastases that may contribute to disease recurrence, is difficult to predict. It may be that a more extensive lymphadenectomy that includes mediastinal nodes provides an advantage, even for type 3 tumors that are generally regarded as being gastric in origin. The lymph nodes and tissue left inside the patient after lymphadenectomy are potentially more important that what is assessed in the pathological specimen; this has been the Achilles heel of previous cancer resection studies assessing the prognostic impact of lymphadenectomy or surgical approach. Future studies, both observational cohort studies and randomized controlled trials, must seek to address this, with pictures or videos at the end of the lymphadenectomy, providing an accurate measurement of intraoperative findings and a measure of quality of surgery and lymphadenectomy. The ongoing TIGER study^4^ will provide further evidence into this ongoing debate. Second, the genomic landscape of these cancers will allow risk stratification by risk profile.^5^,^6^ This will allow a more personalised approach in the management of these cancers, owing to inherent limitations within the Siewert classifications. Finally, patients’ quality of life following esophagectomy and gastrectomy remains an important issue. For some patients, quality of life may be an important consideration when weighing up their decision as to which treatment is best for them, warranting a stronger focus in this area in future studies on this topic. Therefore, the ongoing LASOR^7^ study will further add to this to help in decision making. In summary, adequately powered randomized controlled trials with robust surgical quality assurance is the next step to evaluate the prognostic outcomes of these surgical strategies in GEJ cancer.
